# An infrared energy harvester based on radar cross-section reduction of chiral metasurfaces through phase cancellation approach

**DOI:** 10.1038/s41598-021-90886-0

**Published:** 2021-06-01

**Authors:** Muhammad Amin, Omar Siddiqui, Thamer S. Almoneef

**Affiliations:** 1 College of Engineering, Taibah University, Madinah, 41411 Saudi Arabia; 2 Electrical Engineering Department, College of Engineering, Prince Sattam Bin Abdulaziz University, Al-Kharj, 11942 Saudi Arabia

**Keywords:** Optics and photonics, Metamaterials, Microresonators, Nanoparticles

## Abstract

Conventional metasurface absorbers rely on high dissipation losses by incorporating lossy materials. In this paper, we propose a novel mechanism of absorption based on phase cancellation of polarization states of scattered fields emerging from adjacent L-shaped chiral meta-atoms (unit cells). A linearly polarized wave forms helicoidal currents in each meta-atom leading to diagonally polarized radiated waves. When phase cancellation is employed by reorienting four such meta-atoms in a supercell configuration, contra-directed chiral currents flow in adjacent cells to cancel all the radiated fields in far-field region leading to a minimal broadside radar cross-section. From the reciprocity, the currents that are induced in the meta-atoms produce a null towards the incident direction which can be utilized for infrared energy harvesting. Full wave electromagnetic simulation indicates near perfect resonant absorption around 52.2 THz frequency. Enhanced bandwidth is shown by adding smaller resonators inside the supercell in nested form leading to dual band absorption at 45.2 THz and 53.15 THz.

## Introduction

The constant depletion of the fossil fuels and the associated environmental effects have been driving the intense efforts to harness alternate renewable and clean sources for energy consumption^1–3^. Many regulatory bodies and organizations all over the world have already devised plans for 100% transition to alternate energy sources by the year 2050^4^. One of the most abundantly available resource is the solar energy which has always been utilized by the mankind to satisfy their thermal needs. The discovery of the photovoltaic effect by Edmond Becquerel in 1839 laid the basis of solar to electrical energy conversion. With the development of silicon-based photovoltaic (PV) cell at Bell Labs in 1954, the efficient electrical usage of the solar energy and its large scale distribution became a strong possibility^5,6^. Sun emits its energy as blackbody radiation whose spectrum covers a wide range of frequencies ranging from ultraviolet to infrared^5,7,8^. Although the solar irradiance peaks in the visible spectrum, the infrared radiation amounts to almost 50% of the total radiated power. Earth has its own infrared spectrum which peaks at 10 μm^3,9^. Unfortunately, this immense amount of power have not been largely utilized due to the complexity in direct conversion to electrical energy in the infrared region^3,10^. Since photovoltaic effect is a quantum phenomenon, only photons that have specific energy levels can be effectively absorbed. Hence the conventional silicon-based p–n junctions do not support infrared radiations and as a result have limited efficiencies. Materials such as the narrow gap inorganic semiconductors and compounds and inorganic quantum wells can be employed but their applications have not been fully explored yet^10^. The efficiency of the PV cells can further increased by reducing the spurious reflection of the incident light which requires a very intricate design involving control of feedback reflection path and dissipation of light energy at nanoscale. A 2-bit coding metasurface is proposed to achieve RCS reduction at THz frequencies^11^. Similarly, double split ring resonator metasurface design is proposed for RCS reduction at THz frequencies^12^. Thin film antireflection coatings (ARC) are proposed to suppress the reflection components and increase absorption inside solar cells. However, the performance of ARC is often limited to certain frequencies and cannot support broadband operation^13–15^. More recently, increased absorption has been achieved by periodically embedding metal nanoparticles in the semiconductor to achieve light trapping. By doing so, the optical path increases which leads to enhanced electron hole pair generation^16^.

**Figure 1:**
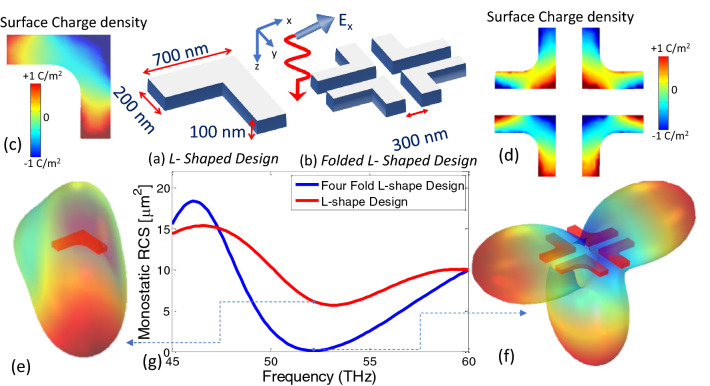
Conceptual demonstration of the RCS reduction (a) L-shaped Unit cell (meta-atom) of the chiral metasurface that resonates at 52.1 THz showing the dimensions and the x-polarized incident electromagnetic field (b) The phase-compensated supercell obtained by four folded L-shaped meta-atoms. (c) Normalized surface charge distribution on single-L unit cell at 52.1 THz. (d) Surface charge distribution on the phase-compensated unit cell showing the oppositely charged dipoles for L elements diametrically opposite to each other causing destructive interference in far-field. (e) The radiation pattern of the single-L unit cell at 52.1 THz showing a large backscatter of incident fields. (f) The radiation pattern for the phase-compensated supercell consisting of four L-shaped elements showing a dramatic reduction of the RCS in direction of incident fields at resonance frequency of 52.1 THz. (g) Spectrum of the monostatic backscattering radar cross section (RCS) of the two structures showing a null for phase-compensated geometry. Illustrations were created in Microsoft Powerpoint 365 [https://www.microsoft.com/en-ww/microsoft-365/powerpoint].

While the photovoltaic cells are based on the particle nature of light, the optical antennas exploit its wave nature for the reception of the solar energy. The antenna along with the rectifying circuitry is termed as *rectenna*^17–19^. Though the theoretical efficiency limit of an optical antenna may reach 100%, the associated nanoscale fabrication have impeded its practical development^20^. With the recent advancement in nanotechnology, the optical rectennas are being considered as strong candidates for future solar energy harvesting, particularly in the infrared spectrum^19–21^. Single resonator elements such as dipole, bow-tie and spiral antennas have been suggested for energy harvesting in the visible and infrared spectra^18,22,23^. An infrared harvesting bow-tie rectenna that employs MIM diodes have experimentally demonstrated^23^. Spiral antennas are considered frequency-independent and have been designed for wideband infrared energy harvesting^24^. To increase the receiving gain which can be needed to drive the rectifier, the antennas are often arranged in the form of an array. The larger array aperture also helps to capture more solar energy. But issues may arise due to the losses in the power combining stage. Infrared arrays have been successfully implemented for energy harvesting and imaging^20,25,26^.

*Metasurface absorber *is a relatively new phenomenon that has become increasingly popular in the field of solar energy harvesting^27–32^. The metasurfaces resemble the antenna arrays in the sense that both are repeated configurations of a unit element. But the two are based on different design principles. In case of an antenna array, the unit element is also an efficient radiator and is separated from the neighbouring element by order of a wavelength. Therefore, the radiation pattern of large arrays approach the array factor and is marked by distinct power nulls^33,34^. On the other hand, the metasurface design is based on collective dispersive response of periodically placed resonators that do not necessarily radiate and are closely located. Consequently, the metasurface reformulates the entire incident wavefront in a manner that is analogous to the wave propagation across a two-medium interface. The metasurfaces in the infrared spectrum are designed by metallic nanoparticles that are smaller than the wavelength and support the localized surface plasmons. The incident electric field excites the electrons in the conduction band leading to the coherent localized plasmon oscillations with a resonant frequency that depends on the geometrical configuration of the nanostructure and the composition of the surrounding material^27,28,35^. In the receiving mode, these trapped plasmon modes can be exploited by either dissipating the incident energy in the form of metallic losses as in absorbers, cloaking and stealth applications^36,37^ or by harvesting it for renewable energy^38^. Since the resonant response strongly depends on the host dielectric material, the plasmonic metasurface have been considered in sensing^39,40^ and photo–detection^41,42^. The recently proposed chiral metasurfaces^43–45^ allow disproportionate absorption of orthogonal electric field components leading to the observance of linear and circular dichroism at THz frequencies. The absorbed portion of the received power can be potentially harvested after the rectification process.

The differential phase angle on an electromagnetic wavefront is an important parameter that defines its orientation and propagation properties. The plane wave expansion methods allows us to express a wavefront in terms of spherical wave coefficients^46^. Conversely, by manipulating the phase of each point on an incident plane wave, the shape and direction of the resulting wavefront can be controlled. By adjusting the phases of adjacent unit cells of a metasurface and by exploiting the plane wave expansion, the incident wavefront has been manipulated in exciting novel applications such as Fresnel reflectors^47,48^, beam steering^49^ and carpet cloaking of objects^50,51^. More recently, the phase control have been digitally encoded by employing switchable unit cells to efficiently manipulate the wavefront of scattered electromagnetic waves^52,53^.

In this paper, we proposed an infrared absorbing metasurface which is designed by exploiting two phenomenon as discussed above, i.e. the partial suppression of orthogonal electric field vector associated with the chiral metasurfaces^43–45^ and the phase manipulation based on the plane wave expansion^46,48,51^ (see Fig. 1). In particular, we tailor the phase response of a metasurface by arranging four chiral L-shaped elements in specific orientations to form a super unit-cell (more commonly termed as a *supercell*). This phase compensating arrangement creates a null in the broadside direction of the metasurface leading to suppression of the radar cross-section (RCS). Since the set-up is used in reflection mode, the reciprocity theorem forces the incident electromagnetic wave to be completely absorbed in the metasurface. The proposed metasurface exploits chirality in conjunction with phase manipulation and reciprocity. Therefore, the phase cancellation takes place on the unit cell basis and without the requirement of an extra absorbing layer as in^54^ which employs magnetic absorbing materials inside a ultrathin metasurface. With our approach, contra-directional chiral currents flow in adjacent unit cells that completely suppress the RCS with minimum dissipative losses. In another contemporary work^55^, the RCS reduction was accomplished by phase mixing from several two-layer super lattices with randomized arrangement. Hence the phase cancellation is a result of superposition of radiations from supercells arrays of different unit cell configurations sharing the same plane. These metasurfaces require more complex fabrication process and have larger apertures.

We show by full-wave simulations a significant reduction of RCS and hence electromagnetic energy absorption of all polarizations in the spectral range of 40 to 60 THz. Multiband absorbing modes can also be obtained by either incorporating more resonant structures of different dimensions in the same supercell or by devising larger supercell arrays. The proposed metasurface support absorption through destructive interference without dissipative losses and can be potentially exploited in infrared energy harvesting applications.

## Design and simuations

### RCS suppression by phase cancellation: unit cell analysis

The RCS suppression is obtained by applying phase cancellation to the metasurfaces that have been designed to be optically active in THz range^44,45^. Consider once again the conceptual Fig. 1. The basic chiral unit cell (or meta-atom), given in Fig. 1a, consists of a L-shape structure designed with 100 nm thick silver material. It should be noted that typically the fabrication tolerances doesn’t allow sharp features for plasmonic nanostructures. Therefore, the right-angle edge of L-shape structure is rounded by a fillet of specific arc radius. The default value for fillet radius is 200 nm unless otherwise stated. By embedding the unit cell in background silica material, its dimensions are optimized so that the unit cell resonates at 54 THz. The phase-compensated version of the unit cell (i.e. the supercell) is obtained by flipping the single element and copying it three times in a 2 × 2 four fold rotational symmetric structure shown in Fig. 1b. To demonstrate the RCS reduction, we compare the scattering characteristics of the two unit cells by illuminating them (numerically) with a y-polarized plane wave using full-wave electromagnetic simulation tool COMSOL. The unit-cells are terminated in radiation boundary conditions. The resulting surface currents and the far-field radiation patterns are then explained. Consider in Fig. 1c,d the normalized surface charge densities which are evaluated by applying the point form Gauss’s Law on the two unit cells. Note that for the single-L, negative charges are accumulated around the center and positive charge are distributed around the corners. By invoking the moments method, it can be well predicted that the two orthogonal dipole moments will emit co- and cross polarized waves which may vectorially add to produce a diagonal or circular polarization in the far-field (depending on the phase difference of the two orthogonal components). This will be further discussed later in the paper. More interestingly, note that the dipole moments formed in the upper two elements of the phase-compensated supercell are exactly out-of-phase with the ones produced in the lower two elements and hence cancel the effect of each other. The phenomenon is more clearly observed in the backscattered radiation patterns considered in Fig. 1e,f. The single L-shaped unit cell supports large backscattered fields which indicates the large reflection coefficient. In comparison, the null in the radiation pattern of the phase-compensated supercell towards the incident direction show the significant reduction in the RCS. A quantitative comparison of the backscattering fields can be obtained by calculating the RCS of the two unit cell configurations from the well-known equation^56^:

1 $$\begin{aligned} \sigma = 4\pi R^2 \frac{|E_{s}|^{2}}{|E_{i}|^{2}}, \end{aligned}$$

where *R* is the distance of the far-field point from the target at which RCS is calculated, $|E_{s}|$ is the normalized back scattered far field and $|E_{i}|$ is the normalized incident field. The monostatic RCS in Fig. 1g is found using back scattered far field $|E_{s}|$ fields for the four-fold and single L-shaped designs. As depicted in Fig. 1g, a minima at 52.1 in the RCS curve of the phase-compensated supercell corroborates the null observed in the radiation pattern. Although, the supercell has almost four times physical cross section compared to the single-L configuration, the corresponding monostatic RCS is suppressed to 0.13 $\upmu \text{m}^2$ which is about 50 times reduction from its value of 6.3 $\upmu \text{m}^2$ for the single-L unit cell at the same frequency.

**Figure 2:**
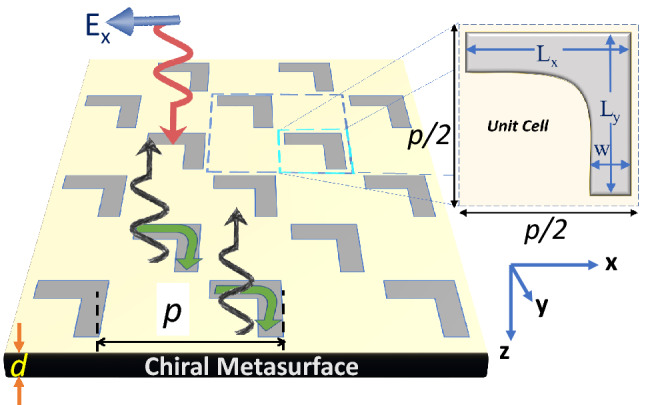
Schematic illustration of plasmonic single-L chiral reflecting metasurface on dielectric substrate of thickness $d=642\,\text{nm}$. Inset shows L-shaped silver nanostructure of thickness 100 nm. Here p = 2 μm, $L_{x}=L_{y}$=700 nm and w = 200 nm. Illustrations were created in Microsoft Powerpoint 365 [https://www.microsoft.com/en-ww/microsoft-365/powerpoint].

### The plasmonic metasurface: periodic analysis

In the previous section, unit cell analysis was used for the conceptual demonstration of the RCS reduction by phase-compensation. In this section, we apply the Floquet boundary conditions to show the phase-compensation phenomenon in infinitely-extended metasurfaces. We employ the Jones Calculus which is a convenient method to analyze the polarization conversion in optically active materials and chiral metasurfaces^44,45,57^. Defining the ($E_{xi}$,$E_{yi}$) and ($E_{xr}$,$E_{yr}$) as the x- and y- directed incident and reflected electric field components, the Jones matrix equation can be written as:

2 $$\begin{aligned} \left( {\begin{array}{*{20}{c}} {{E_xr }}\\ {{E_yr }} \end{array}} \right) = \left( {\begin{array}{*{20}{c}} {{R_xx }}&{}{{R_xy }}\\ {{R_yx }}&{}{{R_yy }} \end{array}} \right) \left( {\begin{array}{*{20}{c}} {{E_xi }}\\ {{E_yi }} \end{array}} \right) \, , \end{aligned}$$

where, $$R_xx $$ and $$R_yy $$ denote the co-polarized, and $$R_xy $$ and $$R_yx $$ represent the cross-polarized reflection coefficients. If transmitted fields are prohibited by placing a perfect conducted towards the transmission direction, the normalized absorption can be calculated by using reflection coefficients as follows:

3 $$\begin{aligned} A = 1-|R_xx |^2-|R_yx |^2, \end{aligned}$$

The reflection properties for the single-L and phase-compensated metasurfaces are further discussed in the following two sub-sections.

### The single-L chiral metasurface

Consider the infinitely extended single-L metasurface in Fig. 2, obtained by periodically repeating the unit-cell with a period *p* equal to 2 μm. Note that a larger period is selected and unit cells are replicated in a checkerboard pattern to avoid the mutual coupling. Also evident in Fig. 2 is the silica substrate of thickness $d =642$ nm which serves as the background material that holds the metallic unit cell. The substrate is backed up by perfectly reflecting ground reflector so that no transmission occurs and all the waves are either absorbed or reflected. In the full-wave simulations implemented in COMSOL software, perfect electric conductor (PEC) boundary is applied in z-direction to simulate the ground; and Bloch-Floquet conditions are applied in x- and y-directions to obtain the infinitely extended metasurface. It is emphasized that the ground plane can be constituted with copper substrate (i.e., finite conductance) that provides negligible variation in overall absorption spectrum when compared to PEC boundary.

**Figure 3:**
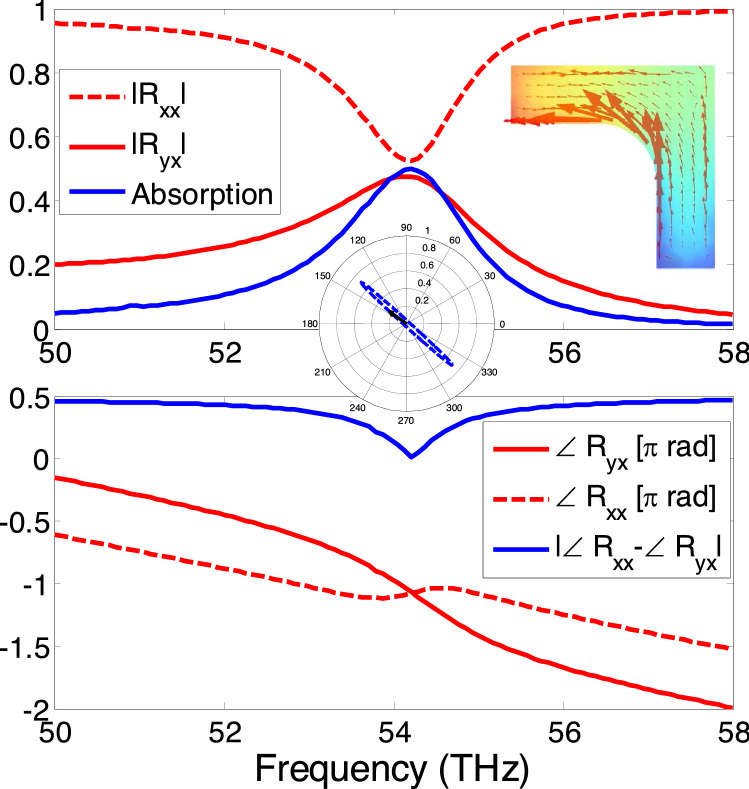
Magnitude and phase of the co-polarized ($R_{xx}$) and cross-polarized ($R_{yx}$) reflection coefficients and the absorption coefficient for the single-L chiral metasurface shown in Fig. 2 when it is illuminated with a x-polarized normally incident electromagnetic wave. The absorption coefficient A is calculated from Eq. 3. The inset shows the magnitude and direction of the surface current density at resonant frequencies of 54.2 THz and the associated polarization ellipse for the reflected fields. The chiral current distribution is provided in instantaneous time varying form in supplementary materials.

By assuming x-polarized electromagnetic wave illuminating the metasurface, the resulting co- and cross polarized reflection coefficients are calculated and are displayed in Fig. 3, along with the absorption coefficient *A*. It is interesting to note that the x-polarized incident field can be partially transformed into the y-directed reflected fields by the localized surface plasmon resonance current. This is possible due to the Fabry Perot resonances that arise due to interference originating from multiple reflections between metasurface and ground plane. As a result anti-clockwise rotational currents are excited in the silver L-shape nanoparticle (see the inset of Fig. 3) at the designed resonance of 54.2 THz. The resonant orthogonal reflected field components have equal magnitude and are perfectly in-phase i.e., $$\angle R_{yx}$$ = $$\angle R_{xx}$$ = $$-\pi $$. Consequently, the in phase localized surface plasmon currents lead to linearly polarized reflected fields along diagonal axis along 135°, also shown in the inset of Figure 3. Looking at the absorption curve, it can be observed that the orthogonal polarization conversion leads to almost 25% absorption of the incident power which may be attributed to the non-radiative losses inside silver nanostructure. It should be noted that since the metasurface is diagonally symmetric, identical reflection response for the y-polarized incident waves should be expected i.e., $$R_xx =R_yy $$ and $$R_yx =R_xy $$. The L-shaped periodic elements can be reoriented to suppress overall scattered fields components from the metasurface as shown in the following section.

**Figure 4:**
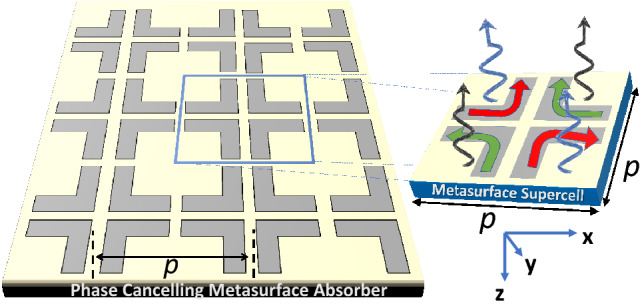
Schematic illustration of phase-compensated metasurface absorber which is formed by periodic arrangement of the folded four-element supercells. Inset shows the supercell structure and the explanation of the phase cancellation phenomenon. Illustrations were created in Microsoft Powerpoint 365 [https://www.microsoft.com/en-ww/microsoft-365/powerpoint].

**Figure 5:**
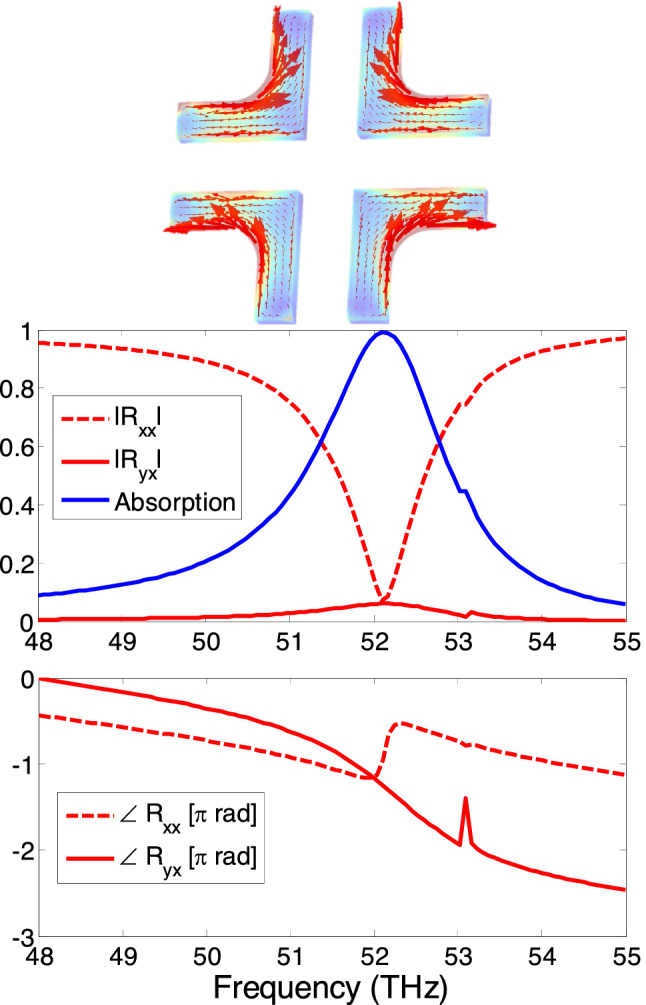
The co- and cross- polarized reflection coefficients of the absorbing phase-compensated metasurface (Fig. 4) along with the absorption spectrum. The inset shows the current density vectors on the supercell at the 52.2 resonance illustrating the vectorial cancellation of diametrically opposite L-elements. The chiral current distribution is provided in instantaneous time varying form in supplementary materials.

### Phase-compensated metasurface absorber

Next we consider the infinitely-extended phase-compensated metasurface shown in Fig. 4, which is obtained by repeating the supercell of Fig. 1b in x and y directions with a periodicity of $p=2\,\upmu \text{m}$. The phase cancelling is qualitatively explained in Fig. 4 by observing the opposite orientation of currents in the diametrically opposite L-elements. Hence by applying the method of moments a complete cancellation of the radiated fields from these surface currents and hence the RCS reduction of Fig. 1 can be expected. To demonstrate the phenomenon, the metasurface is numerically irradiated with a x-polarized electric field and the resulting reflection responses are calculated and are shown in Figure 5. As expected, the co-polarized ($|R_xx |$) and cross-polarized ( $|R_yx |$) reflection coefficients are highly suppressed and therefore, the metasurface exhibits near perfect absorption of the infrared light at around 52.2 THz. A slight shift of resonance from the single-L case (Fig. 4) can be seen which may be attributed to factors like mutual coupling and change in the current distribution. From the inset of Fig. 5, it is clear that the current distribution exhibits mirror symmetry in all the four L-shape nanostructure units that lead to the radiated fields that have complementary phase angles that compensate each other. A comparison of the absorption curves of the single-L and phase-compensated metasurfaces (Figs. 3 and 5), further reveal that the increased absorption is the direct consequence of the destructive interference of these phase-compensated orthogonal electromagnetic fields. The notion of energy harvesting follows from the reciprocity theorem^33^ which dictates the flow of plasmonic surface current (though their vector contribution is zero) to produce the radiation null towards the incident direction. The energy from the current flow in the nanostructures can be tapped through rectification process and harvested. Since the proposed structure based on its symmetrical properties supports absorption for both x- and y- polarized fields, any arbitrarily polarized wave can be demodulated.

**Figure 6:**
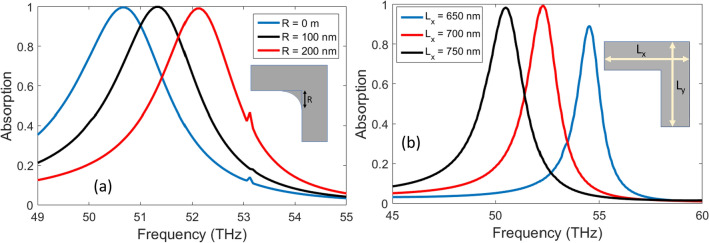
(a) The absorption spectrum due to variation in fillet radius around the right-angle edge of L-shaped structure. (b) The absorption spectrum due to variation in length of L-shaped arms. The graphical description about length of length shaped arms is already provided in Fig. 2.

The phase compensated metasurface absorber can offer resonance tunability due to variation of its geometrical parameters. Typically, the fabrication tolerances doesn’t allow sharp features for plasmonic nanostructures. Therefore, the right-angle edge of L-shape structure is rounded by a fillet of specific arc radius (*R*). The radius of fillet arc (*R*) can affect the spectral absorption as shown in Fig. 6a. The L-shape structure with sharp edge i.e., *R* = 0 supports resonant absorption around 50.7 THz. Due to addition of fillet arc around the edge offers blueshift in the resonance frequency. Therefore, the fillet radii of *R* = 100 nm and *R* = 200 nm offers resonant frequency at 51.4 THz and 52.1 THz respectively.

Similarly, the arm length of L-shaped structure affect the resonant absorption frequency. The effect on spectral absorption characteristics is determined due to variation in arm length ($L_x$ = $L_y$) between 650 nm to 750 nm. It is clear from Fig. 6b that increasing the length arm leads to red shift in the resonance absorption frequency.

**Figure 7:**
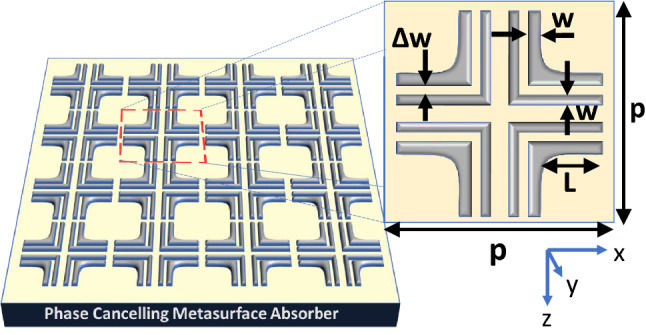
The dual-band phase-compensated metasurface is designed by incorporating two types of resonator structures in the supercell so that there are a total number of eight L-shaped structures. The length of the smaller arms (L) is 512 nm, w = 112 nm and $\Delta w$ = 75 nm. The dimensions of the larger L elements are similar to the ones given in the caption of Fig. 2. Illustrations were created in Microsoft Powerpoint 365 [https://www.microsoft.com/en-ww/microsoft-365/powerpoint].

**Figure 8:**
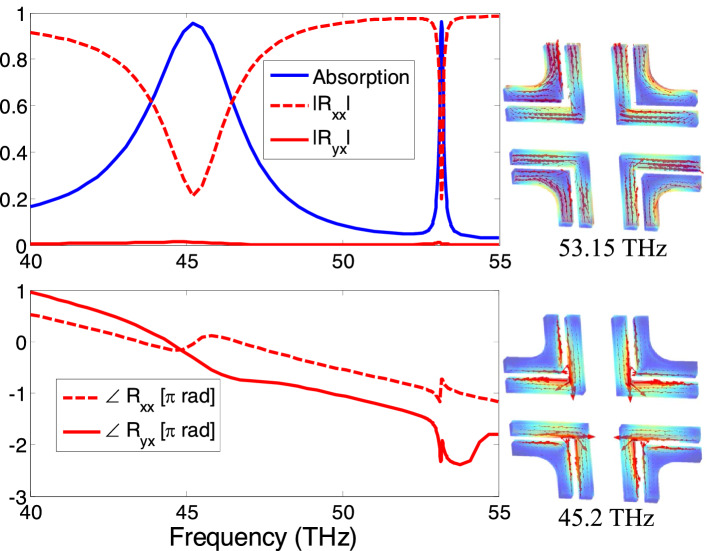
The reflectance and the absorption spectra of the phase-compensated dual band absorber metasurface. The insets show the surface current density vectors at the resonant frequencies of 53.15 THz and 45.2 THz corresponding to the resonances of the larger and smaller L-shaped structures, respectively. The chiral current distribution is provided in instantaneous time varying form in supplementary materials.

### Multi-band phase-compensated metasurface absorber

The infrared blackbody radiation spectra of sun and the cooler earth cover a large bandwidth extending from wavelengths ranging from $0.7\,\upmu \text{m}$ to about $40\,\upmu \text{m}$ which correspond to frequencies between 8 and 430 THz^5,7,8^. The absorption bandwidth of the phase-compensated metasurface can be further increased by inserting more resonators in the supercell so that multiple infrared bands can be absorbed. To accomplish the detuned resonator design, we insert four smaller L-shaped nanostructures in the supercell in nested configuration, as shown in Fig. 7. The supercell is optimized to radiate at 45.2 and 53.15 THz for smaller arms’ lengths (L) equal to 512 nm which are displaced from the longer L arms by $\Delta w=75\,\text{nm}$.

With the x-polarized normal incidence, the spectral responses of the reflection coefficients for the nested supercell metasurface are given in Figure 8. It is clear metasurface supports two absorption modes at the designed infrared frequencies and therefore the absorption efficiency of the metasurface considerable increases. The resonance and the phase compensation mechanism are illustrated in the inset of Fig. 8 with the help of surface current plots. At 45.2 THz, strong localized surface plasmon currents originate on the larger L-shape elements which are oppositely oriented for the diametrically opposite arms. Similarly, the smaller L-shape resonators support intensive localized surface plasmon (counter-directed) currents in the second narrow band absorption mode at 53.15 THz. Following a similar approach, more absorption bands can be added by either nesting more resonators in the same cell or by designing larger multi-resonant supercells. Finally, the shift in the resonant frequency of the lower frequency mode from 52 THz in the original supercell to 45 THz in the nested configuration can be attributed to the larger number of metallic inclusions in the same overall space that may affect the dielectric properties of the background material. It may also be caused by the mutual coupling which can effectively increase the size of the resonators pushing the frequencies towards the red light.

## Conclusion

We propose a phase cancellation approach to reduce the radar cross section (RCS) of infrared chiral metasurfaces so that they behave as highly absorbing energy harvesters. The absorber design is based on the L-shape silver resonating nanostructure which supports localized surface plasmon current in lower infrared frequencies of 40 to 60 THz range. With a single L-shape unit cell, the metasurface is optically active and converts a x- or y-polarized incident wave to a diagonally polarized wave. The phase-compensated metasurface is designed by arranging four L-shape elements in a four-fold rotational symmetry to form a supercell. The incident x-polarized wave induces surface plasmon currents which form contra-directional rotational patterns cancelling the effect of each other. Consequently, two reflected waves having diagonally opposite polarizations are radiated which destructively interfere in the far field causing a significant RCS reduction leading to perfect absorption of incident electromagnetic radiation around 52.2 THz frequency. The rotational currents formed on the plasmonic unit cells can be rectified and utilize in energy harvesting. The absorption efficiency can be considerably improved adding more resonators in the supercell. It is shown that by nesting eight resonators in a supercell, a dual band metasurface absorber can be obtained which supports absorption bands at 45.2 THz and 53.15 THz frequencies. The proposed metasurface is based on destructive far field interference and hence does not require lossy materials to completely absorb the electromagnetic wave. Hence the phase compensation method offers efficient infrared energy harvesting at low cost while supporting minimum energy loss. Since the propose metasurface is optically active, it can demodulate several combinations of polarizations leading to higher efficiency compared to singly polarized structures.

## Methods

### Full wave electromagnetic simulations

The metasurface response was investigated with Full wave three dimensional simulations in COMSOL Multiphysics software. The supercell was simulated with periodic boundary conditions to mimic the effect of infinitely periodic cells along x- and y- directions. The optical material properties for silver nanostructure was obtained by Rakić model for thin metallic films^58^. The fully reflecting ground plane was assumed as perfectly reflecting conductor material with sufficiently large thickness compared to skin depth of metal. The full reflecting characteristics of ground plane provide the feedback path for resonant reflection and enables zero transmission through the metasurface. The monostatic RCS response in Fig 1g is calculated for normally incident and linearly polarized incident planewave with scattered field formulation. Similarly, the three dimensional radiation pattern in Fig. 1e–f are calculated by integrating the radiated fields on a spherical surface enclosing the meta-atoms in the far-field region. The meta-atoms are embedded in silica material. Surface charge distribution on the surface of meta-atoms is calculated by Gauss’ law in point form.

### Locus of reflected electric field vector in space and time

Polarization ellipse and vector plots of electric field vector orientation are provided in inset of e.g, Fig. 3 at resonance frequency of L-shaped nanostructure. Such plot shows the time domain perspective and ultimately provide better understanding of the polarization phase cancellation response. Here, we explain the method to plot the sketch of electric field in space and time. Complex reflection coefficients $R_{xx}$ and $R_{yx}$ are obtained from Full wave simulations. The total reflected electric field phasor is given as follows:

4 $$\begin{aligned} \widetilde{{R}}(z)=(R_{xx}{\hat{a}}_x+R_{yx}{\hat{a}}_y)e^{-ikz} \end{aligned}$$

The corresponding instantaneous electric field is given as follows:

5 $$\begin{aligned} \vec {{R}}(z,t)=\mathfrak {R}e\left[ \widetilde{{R}}(z)e^{-i\omega t}\right] \end{aligned}$$

We are assuming the reflected port to be in the far field of the metasurface. The locus of reflected electric field is plotted in time using small time steps. Contour plots are built upon the locus of electric field vector tip for a complete time period (T).

### Time varying surface current density

The magnitude and direction of surface current distribution influences the overall reflection characteristics. Therefore, the phasor form of surface current density is another important parameter that explains the polarization phase cancellation characteristics as shown in Figs. 3, 5 and 8. The phasor form of vector components of current density distribution on the supercell are obtained from Full wave simulations. The phasor form of vector components of current density distribution can be transformed into instantaneous form as follows:

6 $$\begin{aligned} \vec {{J}}(t)=\mathfrak {R}e\left[ \left( J_x{\hat{a}}_x+J_y{\hat{a}}_y\right) e^{-i\omega t}\right] \end{aligned}$$

The instantaneous form of surface current density using Eq. 6 is obtained and provided in Figs. 3, 5 and 8. Time varying surface current distribution for Figs. 3, 5 and 8 is provided in supplementary materials.

## Supplementary Information

Supplementary Video 1.

Supplementary Video 2.

Supplementary Video 3.

Supplementary Video 4.

## References

[1] Manzano-Agugliaro F, Alcayde A, Montoya FG, Zapata-Sierra A, Gil C (2013). Scientific production of renewable energies worldwide: An overview. Renew. Sustain. Energy Rev..

[2] Khaligh A, Onar OC (2017). Energy Harvesting: Solar, Wind, and Ocean Energy Conversion Systems.

[3] Mescia L, Massaro A (2014). New trends in energy harvesting from earth long-wave infrared emission. Adv. Mater. Sci. Eng..

[4] Singer, S., Denruyter, J.-P. & Yener, D. The energy report: 100% renewable energy by 2050. In *Towards 100% Renewable Energy*, 379–383 (Springer, 2017).

[5] Smets AH, Jäger K, Isabella O, Swaaij RA, Zeman M (2015). Solar Energy: The Physics and Engineering of Photovoltaic Conversion, Technologies and Systems.

[6] Desideri U, Asdrubali F (2018). Handbook of Energy Efficiency in Buildings: A Life Cycle Approach.

[7] Lerner PB, Cutler PH, Miskovsky NM (2015). Coherence properties of blackbody radiation and application to energy harvesting and imaging with nanoscale rectennas. J. Nanophotonics.

[8] Pan Y, Powell C, Song A, Balocco C (2014). Micro rectennas: Brownian ratchets for thermal-energy harvesting. Appl. Phys. Lett..

[9] Bhatia S (2014). Advanced Renewable Energy Systems, (Part 1 and 2).

[10] Dimitriev O (2019). Harvesting of the infrared energy: Direct collection, up-conversion, and storage. Semicond. Phys. Quantum Electron. Optoelectron..

[11] Liang L (2016). Broadband and wide-angle RCS reduction using a 2-bit coding ultrathin metasurface at terahertz frequencies. Sci. Rep..

[12] Qi Y, Zhang B, Liu C, Deng X (2020). Ultra-broadband polarization conversion meta-surface and its application in polarization converter and RCS reduction. IEEE Access.

[13] Hussain B, Ebong A, Ferguson I (2015). Zinc oxide as an active n-layer and antireflection coating for silicon based heterojunction solar cell. Sol. Energy Mater. Sol. Cells.

[14] Zhao J, Green MA (1991). Optimized antireflection coatings for high-efficiency silicon solar cells. IEEE Trans. Electron Devices.

[15] Bilyalov RR, Stalmans L, Schirone L, Levy-Clement C (1999). Use of porous silicon antireflection coating in multicrys talline silicon solar cell processing. IEEE Trans. Electron Devices.

[16] Mohsin AS, Mobashera M, Malik A, Rubaiat M, Islam M (2020). Light trapping in thin-film solar cell to enhance the absorption efficiency using FDTD simulation. J. Opt..

[17] Corkish R, Green M, Puzzer T (2002). Solar energy collection by antennas. Sol. Energy.

[18] González F, Boreman G (2005). Comparison of dipole, bowtie, spiral and log-periodic IR antennas. Infrared Phys. Technol..

[19] Bharadwaj P, Deutsch B, Novotny L (2009). Optical antennas. Adv. Opt. Photonics.

[20] Sabaawi, A. M., Tsimenidis, C. C. & Sharif, B. S. Overview of nanoantennas for solar rectennas. In *Rectenna Solar Cells*, 231–256 (Springer, 2013).

[21] Moddel G, Grover S (2013). Rectenna solar cells.

[22] El-Toukhy YM (2016). Optimized tapered dipole nanoantenna as efficient energy harvester. Opt. Express.

[23] Gadalla MN, Abdel-Rahman M, Shamim A (2014). Design, optimization and fabrication of a 28.3 thz nano-rectenna for infrared detection and rectification. Sci. Rep..

[24] Wang K (2016). Design and analysis of a square spiral nano-rectenna for infrared energy harvest and conversion. Opt. Mater. Express.

[25] González FJ, Ilic B, Alda J, Boreman GD (2005). Antenna-coupled infrared detectors for imaging applications. IEEE J. Sel. Top. Quantum Electron..

[26] Sabaawi AM, Tsimenidis CC, Sharif BS (2013). Planar bowtie nanoarray for thz energy detection. IEEE Trans. Terahertz Sci. Technol..

[27] Aydin K, Ferry VE, Briggs RM, Atwater HA (2011). Broadband polarization-independent resonant light absorption using ultrathin plasmonic super absorbers. Nat. Commun..

[28] Watts CM, Liu X, Padilla WJ (2012). Metamaterial electromagnetic wave absorbers. Adv. Mater..

[29] Azad AK (2016). Metasurface broadband solar absorber. Sci. Rep..

[30] Katrodiya D, Jani C, Sorathiya V, Patel SK (2019). Metasurface based broadband solar absorber. Opt. Mater..

[31] Amin, M. Nanoplasmonic light trapping metascreen encompassing spectrally dense region of solar spectrum. *Plasmonics* **15**, 861–867. 10.1007/s11468-019-01089-3 (2020).

[32] Alaee R, Albooyeh M, Rockstuhl C (2017). Theory of metasurface based perfect absorbers. J. Phys. D Appl. Phys..

[33] Balanis CA (2016). Antenna Theory: Analysis and Design.

[34] Stutzman WL, Thiele GA (2012). Antenna Theory and Design.

[35] Petryayeva E, Krull UJ (2011). Localized surface plasmon resonance: Nanostructures, bioassays and biosensing—A review. Anal. Chim. Acta.

[36] Iwaszczuk K (2012). Flexible metamaterial absorbers for stealth applications at terahertz frequencies. Opt. Express.

[37] Jeong H, Nguyen TT, Lim S (2018). Meta-dome for broadband radar absorbing structure. Sci. Rep..

[38] Muhammad N (2018). Plasmonic metasurface absorber based on electro-optic substrate for energy harvesting. Materials.

[39] Liu N, Mesch M, Weiss T, Hentschel M, Giessen H (2010). Infrared perfect absorber and its application as plasmonic sensor. Nano Lett..

[40] Amin M, Siddiqui O, Abutarboush H, Farhat M, Ramzan R (2021). A thz graphene metasurface for polarization selective virus sensing. Carbon.

[41] Dao TD (2016). Hole array perfect absorbers for spectrally selective midwavelength infrared pyroelectric detectors. ACS Photonics.

[42] Jing YL (2016). Pixel-level plasmonic microcavity infrared photodetector. Sci. Rep..

[43] Hu J (2017). All-dielectric metasurface circular dichroism waveplate. Sci. Rep..

[44] Amin M, Siddiqui O, Farhat M (2020). Metasurface supporting broadband circular dichroism for reflected and transmitted fields simultaneously. J. Phys. D Appl. Phys..

[45] Amin M, Siddiqui O, Farhat M (2020). Linear and circular dichroism in graphene-based reflectors for polarization control. Phys. Rev. Appl..

[46] Clemmow PC (2013). The Plane Wave Spectrum Representation of Electromagnetic Fields: International Series of Monographs in Electromagnetic Waves.

[47] Fan Q (2017). Visible light focusing flat lenses based on hybrid dielectric-metal metasurface reflector-arrays. Sci. Rep..

[48] Amin M, Siddiqui O, Farhat M, Khelif A (2018). A perfect fresnel acoustic reflector implemented by a fano-resonant metascreen. J. Appl. Phys..

[49] He M (2020). Metasurface-based wide-angle beam steering for optical trapping. IEEE Access.

[50] Hsu, L., Lepetit, T. & Kanté, B. Extremely thin dielectric metasurface for carpet cloaking. *Prog. Electromagn. Res.* **152**, 33–40 (2015).

[51] Amin M, Siddiqui O, Orfali W, Farhat M, Khelif A (2018). Resonant beam steering and carpet cloaking using an acoustic transformational metascreen. Phys. Rev. Appl..

[52] Ma Q, Cui TJ (2020). Information metamaterials: bridging the physical world and digital world. PhotoniX.

[53] Wu RY (2019). Digital metasurface with phase code and reflection–transmission amplitude code for flexible full-space electromagnetic manipulations. Adv. Opt. Mater..

[54] Li W (2019). Broadband radar cross section reduction by in-plane integration of scattering metasurfaces and magnetic absorbing materials. Results Phys..

[55] Yang J, Huang C, Song J, Ji C, Luo X (2019). Ultra-broadband low scattering metasurface utilizing mixed-elements based on phase cancellation. J. Phys. D Appl. Phys..

[56] Knott EF, Schaeffer JF, Tulley MT (2004). Radar Cross Section.

[57] Fowles GR (1989). Introduction to Modern Optics.

[58] Rakić AD, Djurišic AB, Elazar JM, Majewski ML (1998). Optical properties of metallic films for vertical-cavity optoelectronic devices. Appl. Opt..

